# Artificial Intelligence in Orthopaedics: Current Evidence and Clinical Translation Across the Patient Care Pathway

**DOI:** 10.3390/jcm15176552

**Published:** 2026-08-25

**Authors:** Rafael De Nigris González, Priscila Luiza Mello

**Affiliations:** 1 Hospital Nove de Julho, São Paulo 01409-002, Brazil; 2 Docente no Programa de Pós Graduação em Enfermagem, Universidade Guarulhos (UnG), São Paulo 07023-070, Brazil; priscila_mello@msn.com

**Keywords:** artificial intelligence, clinical decision support, deep learning, implementation science, machine learning, musculoskeletal imaging, orthopaedics

## Abstract

**Background:** Artificial intelligence (AI) has expanded rapidly across orthopaedic practice, yet routine clinical adoption remains limited despite strong technical performance. This narrative review examines why a persistent gap separates technical maturity from clinical maturity across the orthopaedic patient care pathway. **Methods:** We performed a structured qualitative evidence synthesis of contemporary high-level evidence (systematic reviews, diagnostic test accuracy meta-analyses, and structured narrative reviews) retrieved from PubMed/MEDLINE, Scopus, and Web of Science, supplemented by backward screening of reference lists, covering January 2022 to June 2026. Twenty-one evidence syntheses were analysed thematically across six predefined analytical domains and organized according to the orthopaedic patient pathway. Reporting followed the SANRA (Scale for the Assessment of Narrative Review Articles) criteria. **Results:** Musculoskeletal imaging and fracture detection represented the most mature domains, with several applications reaching early clinical adoption. Applications in arthroplasty planning, shoulder surgery, perioperative prediction, multimodal AI, and clinical decision support remained at developing or emerging stages. Recurrent barriers included limited external validation, dataset heterogeneity, poor workflow interoperability, limited explainability, regulatory and ethical uncertainty, and scarce patient-centred outcome evidence. **Conclusions:** The principal challenge facing orthopaedic AI is no longer algorithm development but clinical translation. We propose the ORION Clinical Readiness Framework, an evidence-informed five-domain model describing the transition from Technical Performance through Clinical Validation, Workflow Integration, and Patient Benefit to Routine Clinical Adoption, to guide implementation and future research.

## 1. Introduction

Musculoskeletal disorders represent one of the leading causes of disability worldwide. According to the World Health Organization, more than 1.71 billion people live with a musculoskeletal condition worldwide. The absolute burden has increased over recent decades, driven principally by population growth and population ageing rather than by rising age-standardised rates [1].

Although the absolute number of people affected by musculoskeletal conditions has increased substantially in recent decades, this trend should not be interpreted as a uniform increase in the incidence of every individual disease. The observed growth in absolute burden is largely related to population growth and ageing, while age-standardised rates vary according to the specific condition, population, and geographical region [1].

Importantly, the burden of musculoskeletal disease is not uniform across populations, as sex- and gender-related differences influence the prevalence, presentation, progression, and treatment response of orthopaedic conditions. Females are disproportionately affected by several conditions, including osteoporosis, osteoarthritis, adolescent idiopathic scoliosis, hallux valgus, anterior cruciate ligament injuries, and certain fragility fractures, whereas males may experience greater severity, complication rates, or mortality in selected conditions, including hip fractures and some traumatic injuries. These differences reflect the combined effects of biological factors, such as hormonal regulation, bone and cartilage morphology, and biomechanics, as well as gender-related behavioural, social, and healthcare determinants. Recognition of these differences is particularly relevant to the development and implementation of artificial intelligence systems, as under-representation or inadequate stratification by sex and gender may contribute to biased datasets, reduced model generalizability, and unequal clinical performance [2].

Against this background, artificial intelligence (AI) has emerged as one of the most transformative technologies in contemporary orthopaedics. Advances in machine learning, deep learning, computer vision, natural language processing, multimodal learning, and foundation models have enabled AI systems to analyse complex clinical and imaging datasets with unprecedented speed, consistency, and scalability. Over the past five years, AI applications have expanded rapidly across virtually every orthopaedic subspecialty, including musculoskeletal imaging, fracture detection, arthroplasty, spine surgery, trauma, sports medicine, rehabilitation, oncology, and clinical decision support [3,4,5,6].

Among all current applications, musculoskeletal imaging represents the most mature domain of AI implementation. Multiple systematic reviews and diagnostic meta-analyses have consistently demonstrated that deep learning algorithms achieve diagnostic performance comparable to experienced musculoskeletal radiologists for fracture detection, image segmentation, implant recognition, osteoarthritis grading, and automated image interpretation [7,8,9,10,11]. However, direct comparisons varied substantially across studies, and a single pooled percentage of agreement could not be estimated because of differences in populations, imaging modalities, reference standards, and reported performance metrics. Thus, the available evidence supports comparable performance in selected tasks but does not establish uniform equivalence or superiority across all orthopaedic applications. Beyond imaging, AI has progressively expanded into preoperative planning, patient selection, implant templating, robotic surgery, outcome prediction, postoperative surveillance, and rehabilitation. Although these technologies consistently demonstrate excellent technical performance, considerably fewer studies have evaluated their real-world effectiveness, external validation, workflow integration, or impact on patient-centred outcomes [3,12,13,14,15,16,17,18].

Consequently, an important discrepancy has emerged between technical maturity and clinical maturity. While algorithms continue to improve rapidly, routine implementation into orthopaedic practice remains relatively limited because successful clinical translation depends on factors extending well beyond algorithmic performance, including interoperability, explainability, ethical governance, clinician acceptance, regulatory approval, and demonstration of meaningful patient benefit [3,5,6,11,17,19,20].

Most contemporary reviews have focused on isolated AI applications or individual orthopaedic subspecialties [3,4,5,6,17,18,19]. Reviews dedicated to musculoskeletal imaging primarily emphasize diagnostic accuracy and image-analysis tasks [7,8,9,10,11]; arthroplasty-related reviews address surgical indication, implant-related imaging, and outcome prediction [13,15,20]; trauma reviews evaluate fracture detection and machine-learning prediction models [7,8,14,21,22]; and shoulder surgery reviews assess disease-specific applications, including shoulder arthroplasty and outcome prediction [13,16]. Although these studies provide valuable insights into individual technologies, few examine AI as a continuous translational process spanning the entire orthopaedic patient journey [3,4,5,6,17,18,19].

Therefore, the present review adopts a broader translational perspective. Rather than asking whether AI algorithms achieve high diagnostic performance, we investigate why AI has not yet achieved widespread routine clinical implementation in orthopaedics despite remarkable technological progress. Through a structured qualitative evidence synthesis of contemporary high-level evidence, we organized current knowledge according to the orthopaedic patient pathway, identified the principal barriers limiting implementation, synthesized recommendations proposed across the literature, and developed the Orthopaedic Readiness for the Implementation of AI in Practice (ORION) Clinical Readiness Framework, an evidence-informed conceptual model describing the sequential transition from Technical Performance to Clinical Validation, Workflow Integration, Patient Benefit, and ultimately Routine Clinical Adoption. We propose that the principal challenge facing orthopaedic AI is no longer algorithm development but successful clinical translation into everyday patient care [3,6,17,18,19].

**Figure 1:**
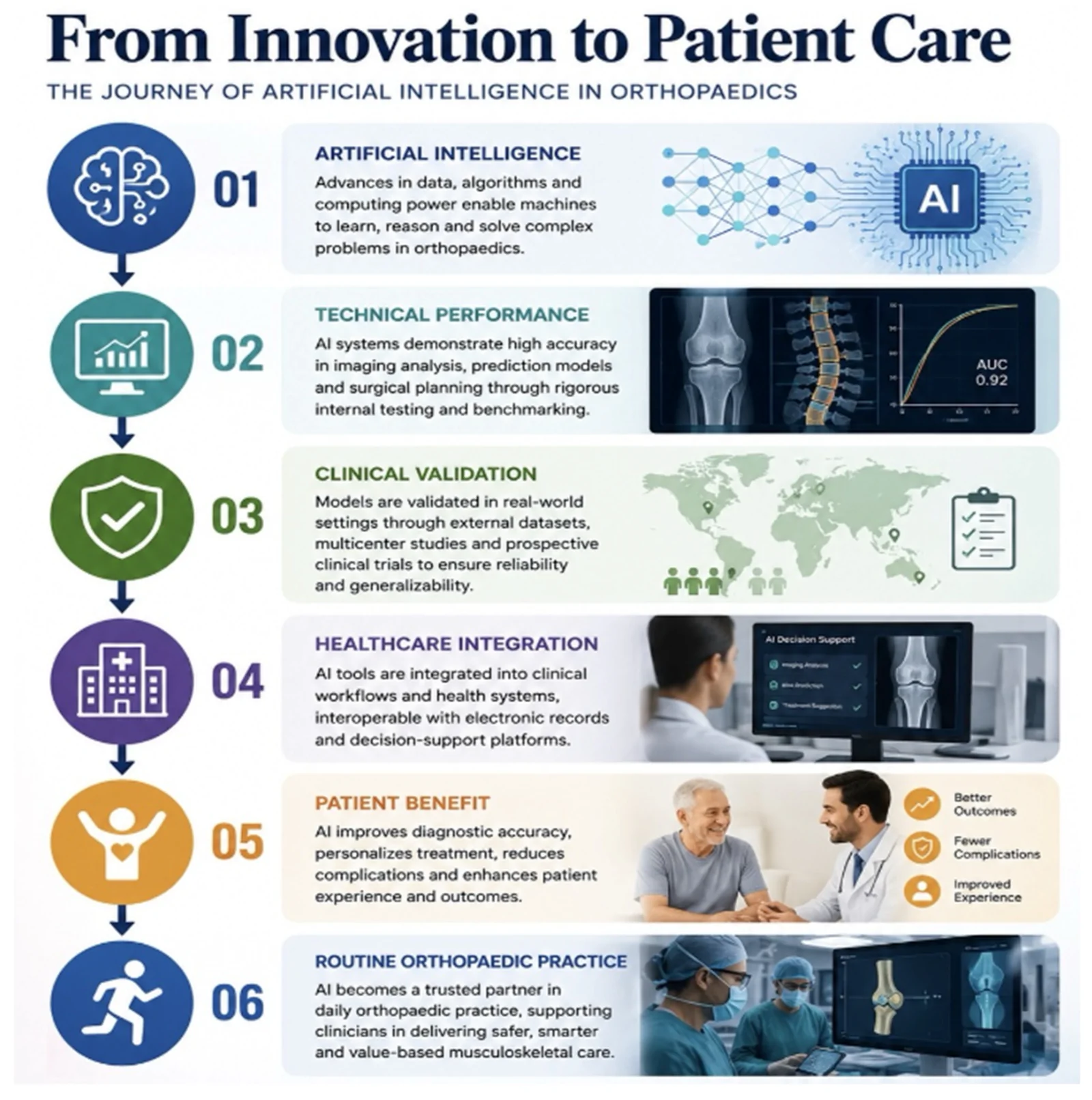
From Innovation to Patient Care: The Journey of Artificial Intelligence in Orthopaedics.

Figure 1 illustrates the broader translational journey of artificial intelligence from technological innovation and algorithm development toward clinical use and patient care. The innovation and algorithm-development phase precedes the five ORION domains. The ORION Clinical Readiness Framework itself, including its operational domains and assessment criteria, is presented separately in Figure 1 and Table 1.

## 2. Methods

### 2.1. Study Design

This study was conducted as a structured qualitative evidence synthesis designed to critically evaluate the clinical translation of artificial intelligence (AI) across the orthopaedic patient care pathway. Given the substantial heterogeneity among AI technologies, orthopaedic subspecialties, study designs, outcome measures, and validation strategies, quantitative pooling was considered inappropriate. Instead, an interpretative synthesis was undertaken to organize the available evidence, identify recurrent implementation patterns, evaluate the maturity of the evidence, and classify the literature according to clinically meaningful translational domains [3,5,17,19].

The conduct and reporting of the narrative synthesis were guided by the Scale for the Assessment of Narrative Review Articles (SANRA). This review was not designed as a formal systematic review, and no quantitative meta-analysis of diagnostic or predictive performance was undertaken. A formal risk-of-bias appraisal of the included studies was also not performed, which is acknowledged as a limitation of the present synthesis.

Unlike conventional narrative reviews that focus on isolated AI applications or individual orthopaedic subspecialties, the present review adopted a translational perspective, examining the progression of AI from technological development and validation toward clinical implementation across the orthopaedic patient care pathway [17,18].

### 2.2. Literature Search Strategy

A structured literature search was performed in PubMed/MEDLINE, Scopus, and Web of Science. PubMed/MEDLINE served as the primary biomedical database, whereas Scopus and Web of Science were searched to capture relevant literature from orthopaedic, engineering, and computer-science fields. Additional publications were identified through backward screening of the reference lists of all included evidence syntheses and other highly relevant systematic and narrative reviews.

The search strategy combined Medical Subject Headings (MeSH) and free-text terms related to artificial intelligence, machine learning, deep learning, convolutional neural networks, computer-aided systems, orthopaedics, musculoskeletal imaging, fracture detection, arthroplasty, spine surgery, sports medicine, trauma, rehabilitation, surgical planning, robotics, and clinical decision support.

The search was restricted to publications dated from 1 January 2022 to 30 June 2026 and to articles published in English. This date range was selected pragmatically to focus the review on recent evidence syntheses addressing the contemporary clinical and translational landscape of artificial intelligence in orthopaedics. The selected period was intended to provide a focused and manageable overview of recent literature, rather than to represent a formally defined period of quantitative growth or to imply that relevant evidence published before 2022 was methodologically unimportant. Studies published before 2022 were not searched or analysed as individual records; when discussed, they were considered only as studies cited within the included evidence syntheses. The language restriction was pragmatic rather than methodological and is acknowledged as a limitation.

The PubMed/MEDLINE search was conducted using the following strategy: (“artificial intelligence”[MeSH] OR “machine learning” OR “deep learning” OR “convolutional neural network*” OR “computer-aided”) AND (“orthopedics”[MeSH] OR orthopaedic* OR musculoskeletal OR fracture* OR arthroplasty OR “spine surgery” OR “sports medicine” OR trauma OR rehabilitation) AND (“systematic review” OR “meta-analysis” OR review) AND (“01/01/2022”[Date—Publication]: “30/06/2026”[Date—Publication]).

Equivalent search strategies, adapted to the indexing systems of Scopus and Web of Science, were also applied. The last database search was performed on 30 June 2026. 

### 2.3. Study Selection and Data Extraction

Titles and abstracts were screened independently by two investigators (R.D.N.G. and P.L.M.), and the full texts of potentially eligible records were assessed according to the predefined eligibility criteria. Duplicate records were removed before screening. Disagreements at any stage were resolved through discussion until consensus was reached.

**Figure 2:**
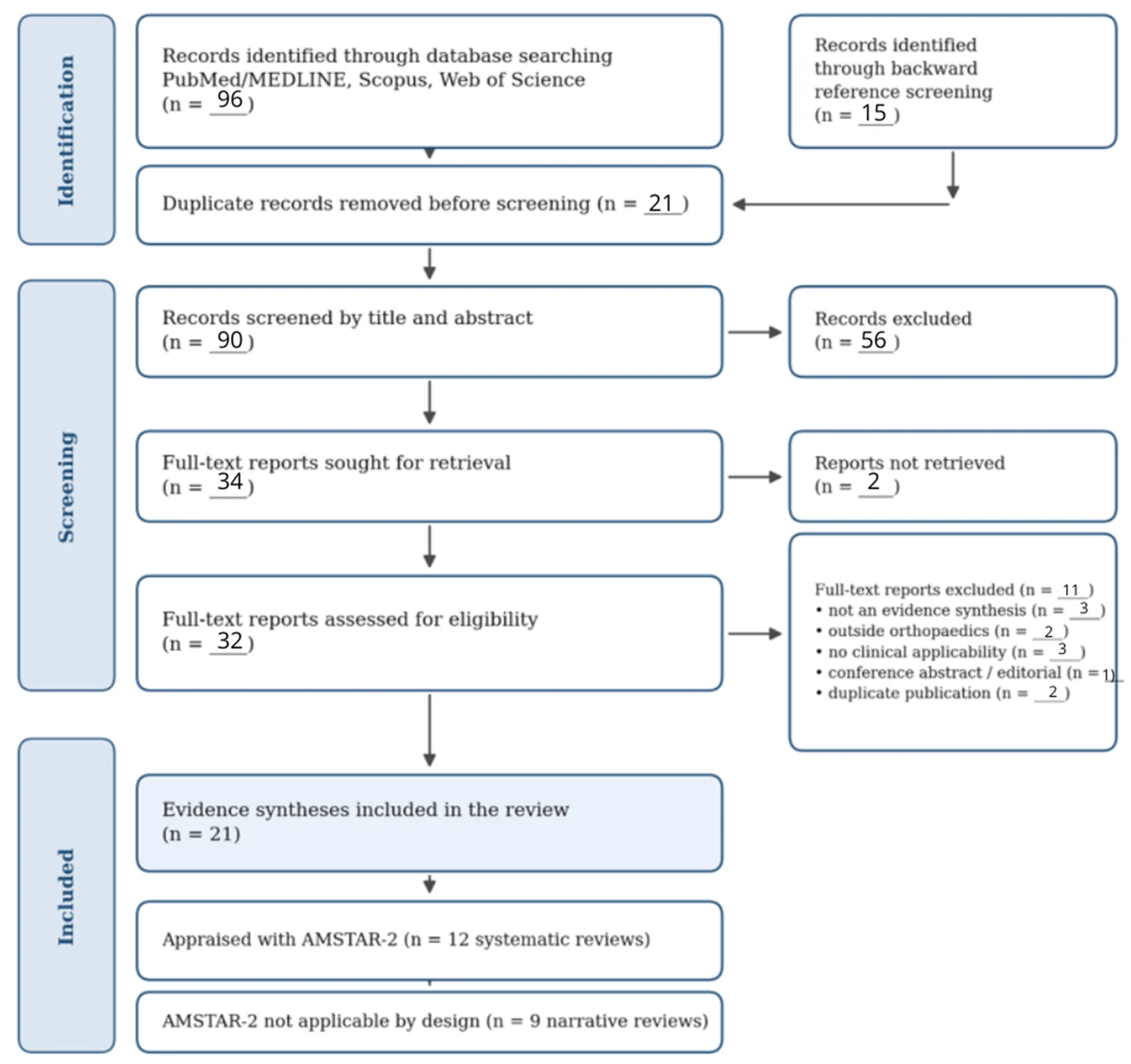
Study-selection flow diagram.

Data extraction was performed independently by the two investigators using a standardized Evidence Extraction Matrix and was subsequently cross-checked. Any discrepancies were reconciled by consensus. The study-selection process and the numbers of records identified, screened, assessed for eligibility, and excluded are summarized in the study-selection flow diagram (Figure 2).

Flow of records through identification, screening, eligibility assessment and inclusion. AMSTAR-2, A MeaSurement Tool to Assess systematic Reviews, version 2.

Appraisal of included syntheses. The methodological quality of the included evidence syntheses was appraised using the AMSTAR-2 instrument, with overall confidence rated as high, moderate, low, or critically low. A formal risk-of-bias appraisal of the primary studies within each synthesis was not undertaken, consistent with the narrative design, and is acknowledged as a limitation.

### 2.4. Eligibility Criteria

Publications were considered eligible if they fulfilled all predefined inclusion criteria. Eligible publications included systematic reviews, systematic reviews with meta-analysis, diagnostic test accuracy meta-analyses, structured narrative reviews, and comprehensive narrative reviews.

Eligible publications were required to evaluate clinically applicable artificial intelligence (AI) technologies in one or more orthopaedic domains, including musculoskeletal imaging, fracture detection, arthroplasty, shoulder surgery, spine surgery, trauma, sports medicine, rehabilitation, robotic surgery, clinical decision support, or the prediction of surgical outcomes.

Only publications written in English were eligible for inclusion. This language restriction was pragmatic rather than methodological and is acknowledged as a limitation.

Conference abstracts, editorials, expert opinion articles, technical algorithm-development studies without clear clinical applicability, duplicate publications, and publications unrelated to musculoskeletal medicine or orthopaedics were excluded. Following the eligibility assessment, 21 contemporary evidence syntheses constituted the final evidence base of the present review.

### 2.5. Data Extraction

Data extraction was performed using a standardized Evidence Extraction Matrix (EEM) specifically developed for this review. The two investigators independently extracted data from each included publication. The extracted information was subsequently cross-checked, and discrepancies were resolved through discussion and consensus.

The following variables were extracted: bibliographic information, publication year, evidence type, orthopaedic subspecialty, primary AI application, stage of the orthopaedic patient care pathway, AI methodologies employed, principal findings, external validation status, workflow integration, evidence of patient benefit, barriers to implementation, and recommendations for clinical translation.

A summary of the extracted evidence is presented in Table 1, allowing direct comparison between publications despite considerable methodological heterogeneity.

### 2.6. Qualitative Evidence Synthesis

Evidence synthesis was performed using a thematic interpretative approach [3,17,19]. Because of the substantial heterogeneity in AI technologies, orthopaedic applications, study designs, outcome measures, and validation strategies, diagnostic accuracy metrics and other quantitative outcomes were not pooled.

Instead, the included evidence syntheses were analyzed according to six predefined domains: (1) characteristics of the evidence base; (2) AI across the orthopaedic patient care pathway; (3) clinical readiness; (4) barriers to clinical translation; (5) recommendations for clinical implementation; and (6) future research priorities [4,5,17].

This approach enabled structured comparison across different AI technologies, imaging modalities, and orthopaedic applications while maintaining an emphasis on clinical translation and implementation rather than isolated algorithmic performance [2,7,11,17].

## 3. Results

### 3.1. Characteristics of the Evidence Base

The database searches identified 96 records, and backward screening of reference lists identified an additional 15 records. After removal of 21 duplicate records, 90 records were screened by title and abstract, of which 56 were excluded. Thirty-four full-text reports were sought for retrieval; 2 reports could not be retrieved, and 32 full-text reports were assessed for eligibility. Of these, 11 were excluded: 3 were not evidence syntheses, 2 were outside the field of orthopaedics, 3 lacked clinical applicability, 1 was a conference abstract or editorial, and 2 were duplicate publications. A total of 21 contemporary evidence syntheses published between 2022 and 2026 were included in the final review. The study-identification and selection process is presented in Figure 2.

The included publications comprised systematic reviews, systematic reviews with meta-analysis, diagnostic test accuracy meta-analyses, structured narrative reviews, and comprehensive narrative reviews addressing clinically relevant applications of artificial intelligence (AI) in orthopaedics. Collectively, these evidence syntheses covered a broad range of orthopaedic applications, including musculoskeletal imaging, fracture detection, trauma, arthroplasty, shoulder surgery, spine surgery, sports medicine, orthopaedic oncology, robotic surgery, rehabilitation, and clinical decision support.

Of the 21 included evidence syntheses, 12 systematic reviews or meta-analyses were appraised using AMSTAR-2, whereas the instrument was not applicable by design to the 9 narrative reviews. The characteristics of the included evidence syntheses are summarized in Table 1.

Most of the included evidence consisted of systematic reviews and meta-analyses, reflecting the increasing methodological maturity of AI research within orthopaedics. Nevertheless, comprehensive narrative reviews remained particularly valuable for rapidly evolving topics such as multimodal AI, explainable AI (XAI), foundation models, robotics, digital twins, and implementation science, where prospective evidence remains limited [3,4,5,17].

Overall, the current literature demonstrates a clear transition from isolated proof-of-concept algorithms toward clinically oriented investigations focusing on implementation, although substantial translational challenges persist [6,18,19].

### 3.2. Applications of Artificial Intelligence Across the Orthopaedic Care Pathway

Analysis of the included studies demonstrated that artificial intelligence applications extend beyond isolated diagnostic tasks and address multiple stages of the orthopaedic patient care pathway [17,18]. 

In this review, the term “orthopaedic patient care pathway” refers to the main sequential stages of care identified in the included literature: diagnosis and imaging, patient selection and risk stratification, preoperative planning, intraoperative support, postoperative monitoring, rehabilitation, and long-term follow-up. The AI applications encompassed image interpretation, fracture detection, segmentation, implant recognition, surgical indication, risk and outcome prediction, three-dimensional reconstruction, implant planning, robotic and navigation-assisted surgery, complication surveillance, wearable monitoring, and rehabilitation support. This terminology describes the organizational scope of the synthesis and does not imply that a single AI system currently operates across all stages or that all applications have achieved routine clinical adoption.

Current applications encompass diagnostic imaging, fracture detection, patient selection, preoperative planning, implant templating, robotic-assisted surgery, intraoperative navigation, postoperative surveillance, rehabilitation, remote monitoring, and the prediction of clinical outcomes.

Musculoskeletal imaging was the primary focus of three of the 21 included evidence syntheses, while three additional syntheses specifically addressed AI-assisted fracture detection. Together, these six imaging-related evidence syntheses represented the largest concentration of evidence in the review [7,8,9,10,11,21]. Across the included evidence syntheses, AI-based systems generally demonstrated high diagnostic performance in fracture detection, automated segmentation, implant recognition, osteoarthritis grading, and image interpretation. However, a single overall numerical comparison was not possible because the reviews evaluated different clinical tasks, imaging modalities, reference standards, and performance metrics. Reported performance should therefore be interpreted as task-specific rather than as a single overall measure of diagnostic effectiveness.

Preoperative planning represented the second largest body of evidence [13,15,16,17,21]. Recent studies demonstrated the growing application of artificial intelligence in automated three-dimensional reconstruction, patient-specific implant selection, surgical simulation, implant-sizing prediction, and risk stratification. These applications were particularly well represented in arthroplasty, trauma, and spine surgery.

Intraoperatively, artificial intelligence applications increasingly incorporated robotics, computer vision, navigation systems, augmented reality, and intelligent workflow optimization [4,5,18]. Although these technologies consistently improved technical precision, relatively few studies demonstrated significant improvements in long-term patient outcomes.

Postoperatively, artificial intelligence applications included complication prediction, implant surveillance, wearable monitoring, gait analysis, personalized rehabilitation, and remote follow-up. Compared with imaging, however, these applications remain substantially less mature and continue to rely predominantly on retrospective observational evidence.

The complete distribution of artificial intelligence applications throughout the orthopaedic patient journey is summarized in Table 2 and conceptually illustrated in Figure 1.

### 3.3. Evidence Landscape of Contemporary Orthopaedic AI

Mapping the current literature revealed a markedly asymmetric distribution of evidence across orthopaedic AI applications.

**Figure 3:**
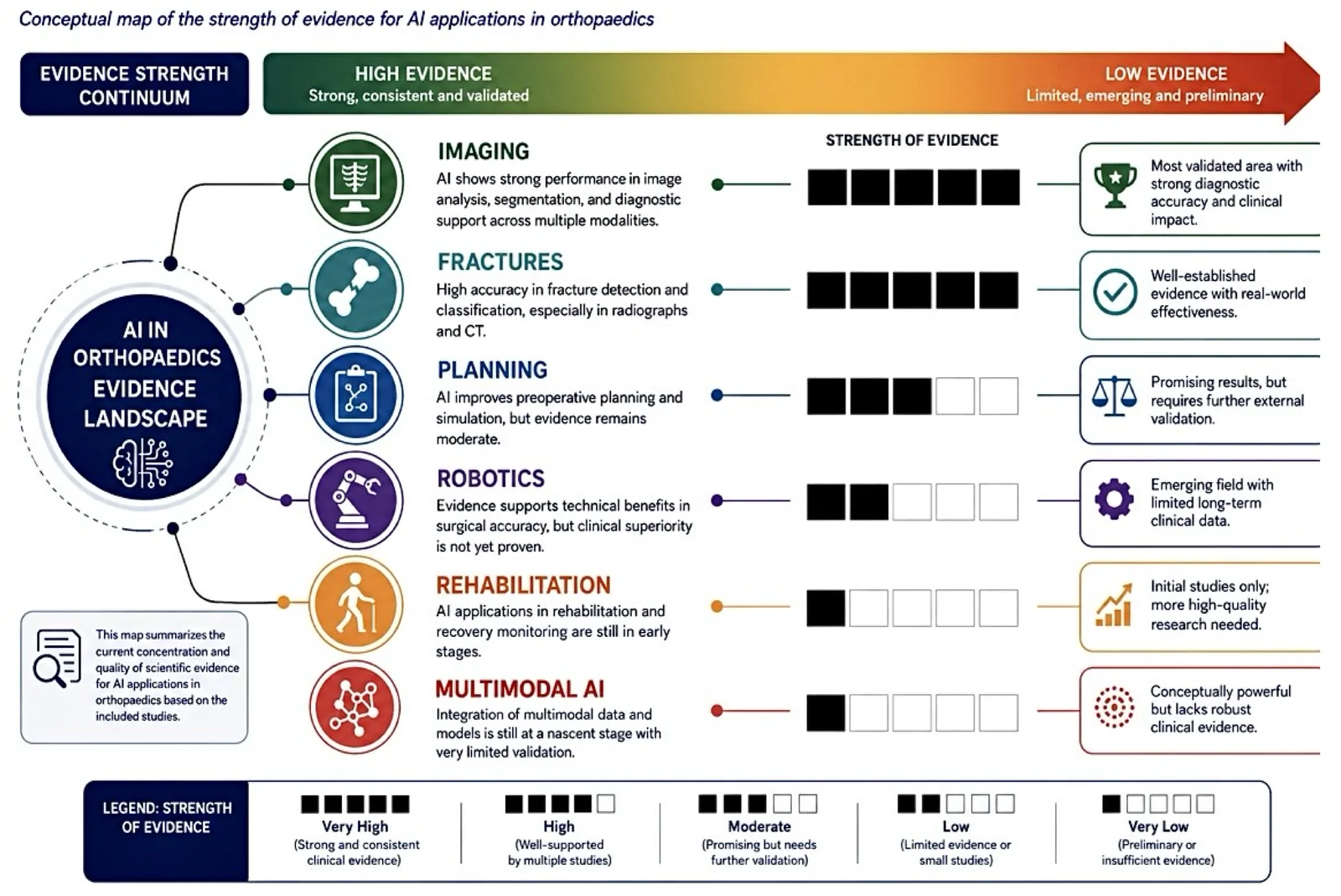
Evidence Landscape of Artificial Intelligence Applications in Orthopaedics.

As illustrated in Figure 3, the current evidence is predominantly concentrated in musculoskeletal imaging, with fracture detection representing the most extensively studied application and the area with the highest concentration of mature evidence [7,10,11]. These domains have benefited from standardized imaging protocols, objective reference standards, large annotated datasets, multiple systematic reviews, and quantitative meta-analyses. Consequently, imaging applications have demonstrated the highest level of clinical maturity.

Applications involving arthroplasty planning, surgical navigation, robotics, and perioperative prediction have demonstrated an intermediate degree of maturity [13,15,16,17]. Although these technologies have shown encouraging improvements in technical performance and operative precision, evidence of corresponding improvements in long-term patient outcomes remains limited [3,4,5,6,12,17,18].

Conversely, rehabilitation, wearable monitoring, multimodal artificial intelligence, foundation models, digital twins, and integrated clinical decision-support systems represent the least mature areas of contemporary research [4,6,18]. Most available studies in these domains remain proof-of-concept investigations or early implementation reports with limited prospective validation.

Overall, the current evidence landscape indicates that artificial intelligence in orthopaedics remains heavily concentrated in image-based applications, whereas technologies requiring integration into complex multidisciplinary clinical workflows continue to evolve at a slower pace.

### 3.4. Clinical Readiness According to the ORION Framework

The ORION Clinical Readiness Framework should be interpreted as an original, evidence-informed conceptual model generated from the qualitative synthesis of the included literature. It is intended to provide a structured language for describing the reported translational maturity of artificial intelligence (AI) applications in orthopaedics, rather than to function as a validated clinical classification system, prediction tool, or quantitative readiness score. The framework has not undergone external validation, prospective testing, formal psychometric evaluation, or assessment of interobserver reliability.

Application of the ORION framework revealed considerable variation in the reported implementation maturity of AI applications across orthopaedic domains.

As summarized in Table 3, fracture detection was the only domain classified as reaching the Early Clinical Adoption stage. This classification was based on its consistently strong technical performance, evidence from external validation, integration into selected clinical or commercial workflows, and reported diagnostic benefit. Musculoskeletal imaging and commercially available radiological platforms demonstrated high technical and workflow maturity but were classified as Developing because evidence of prospective patient-centred benefit and broader independent validation remained limited [7,8,9,10,11].

These applications demonstrated robust technical performance, evidence of external validation, integration into clinical workflows, and potential to support meaningful improvements in diagnosis.

Applications involving shoulder surgery, arthroplasty planning, perioperative prediction, trauma prognostication, multimodal imaging, and clinical decision support were predominantly classified as Developing [13,15,16,17]. Although algorithmic performance was generally excellent, these technologies frequently lacked multicentre validation, standardized implementation strategies, prospective evaluation, and evidence of patient-centred outcomes.

Emerging technologies, including digital twins, multimodal artificial intelligence, wearable monitoring, and integrated precision orthopaedics, remained within the Emerging stage of the ORION framework [4,6]. Overall, these findings demonstrate that technical excellence alone does not equate to clinical readiness. External validation, effective workflow integration, and evidence of meaningful patient benefit remain important prerequisites for progression toward routine clinical implementation.

These categories represent qualitative descriptors of the evidence reported in the included literature and should not be interpreted as definitive assessments of effectiveness, safety, cost-effectiveness, regulatory readiness, or suitability for routine clinical use.

### 3.5. Barriers to Clinical Translation

Despite consistently encouraging technical performance, virtually every included review identified substantial barriers preventing widespread implementation of AI in routine orthopaedic practice (Table 4) [4,6,17,19].

Across the included evidence syntheses, several barriers to the clinical implementation of artificial intelligence in orthopaedics were recurrently identified, including limited external validation, methodological heterogeneity, challenges related to workflow integration, insufficient patient-centred outcome evidence, limited explainability, regulatory uncertainty, and fragmented healthcare data [3,4,5,6,12,13,14,15,16,17,18,19].

Limited external validation, particularly the scarcity of multicentre prospective evaluations across diverse healthcare settings, was frequently highlighted. Other reported barriers included heterogeneous datasets, inconsistent reporting standards, limited interoperability with hospital information systems, insufficient explainability of AI models, regulatory and ethical concerns, algorithmic bias, and uncertainty regarding clinician trust and acceptance [4,5,6,12,14,16,17,18,19].

Although these barriers were reported across several orthopaedic domains, the available evidence does not establish that they affect all subspecialties equally. Overall, the findings suggest that clinical implementation depends not only on technical performance but also on the successful translation of AI into complex real-world healthcare environments [4,6,17,18,19].

### 3.6. Recommendations for Clinical Implementation

Across the included literature, several implementation priorities emerged consistently (Table 5) [4,6,17,18].

First, AI should initially function as a clinical decision-support tool rather than an autonomous replacement for clinician judgment. Second, broader implementation requires robust external validation, standardized reporting, and prospective evaluation across multiple institutions [6,17]. Third, successful deployment depends on seamless integration into existing clinical workflows, electronic health records, radiology platforms, and surgical planning systems [18,19]. Finally, explainability, ethical governance, regulatory oversight, and continuous post-implementation monitoring were repeatedly identified as prerequisites for sustainable adoption [4,19].

Taken together, these findings suggest that future progress in orthopaedic AI will depend less on incremental improvements in algorithmic performance and more on advances in implementation science, multidisciplinary collaboration, and demonstration of meaningful patient benefit.

## 4. Discussion

### 4.1. The Translation Gap: Why Clinical Adoption Has Lagged Behind Technological Progress

The present review is consistent with previous reviews showing that artificial intelligence has progressed beyond isolated experimental applications and is now being investigated across multiple stages of orthopaedic care [3,4,5,6,17,18]. However, our synthesis extends these observations by demonstrating that this expansion in scope has not been accompanied by equivalent progress in external validation, workflow integration, or evidence of patient benefit. Similar concerns regarding the gap between technical performance and clinical implementation have been reported in previous orthopaedic AI reviews, particularly in surgical planning, predictive modelling, and multimodal applications [4,5,6,16,17,18,19]. Across the 21 evidence syntheses included in this review, AI consistently demonstrated excellent performance in fracture detection, musculoskeletal image interpretation, automated segmentation, implant recognition, surgical planning, robotic assistance, and prediction of postoperative complications [3,4,5,17].

Nevertheless, a remarkably consistent observation emerged throughout the literature. Although algorithmic performance has improved substantially during the last decade, widespread implementation into routine orthopaedic practice remains limited. Most published studies continue to rely on retrospective datasets, internally validated algorithms, or proof-of-concept investigations, whereas prospective multicentre implementation studies remain scarce [6,18,19].

This discrepancy represents the translation gap, which emerged as the principal finding of the present review. Current orthopaedic AI research has largely overcome computational limitations; however, successful implementation now depends on factors extending well beyond diagnostic accuracy [6,17,18].

Across virtually all orthopaedic domains, implementation remains constrained by insufficient external validation, fragmented datasets, heterogeneous reporting standards, limited interoperability with hospital information systems, regulatory uncertainty, ethical concerns, algorithmic opacity, and the absence of robust evidence demonstrating improvements in patient-centred outcomes [4,6,17,19].

Accordingly, the major challenge facing orthopaedic AI is no longer algorithm development but successful clinical translation into routine patient care.

### 4.2. Why Musculoskeletal Imaging Represents One of the Most Advanced Domains of AI Translation

Among the orthopaedic applications evaluated in this review, musculoskeletal imaging represented one of the most advanced domains along the translational pathway, particularly in terms of technical performance and workflow integration [7,8,9,10,11].

This pattern was reported across reviews evaluating fracture detection, implant recognition, osteoarthritis grading, image segmentation, and other diagnostic imaging applications [7,8,9,10,11]. Several factors may help explain this relative maturity.

First, imaging often benefits from relatively standardized acquisition protocols, which may reduce variability across institutions. Second, radiological diagnosis commonly relies on defined reference standards, thereby facilitating supervised learning and quantitative validation. Third, the widespread use of digital imaging infrastructures, including Picture Archiving and Communication Systems (PACS), may facilitate the integration of AI tools into existing radiology workflows [4,5,6,9,11].

Finally, the availability of large annotated imaging datasets has supported the development and evaluation of convolutional neural networks with high diagnostic performance [7,8,9,10,11]. Taken together, these characteristics may explain why musculoskeletal imaging has progressed further along the translational pathway than many other orthopaedic AI applications. However, this relative maturity should not be equated with uniform routine clinical adoption.

Nevertheless, even within imaging, prospective multicentre validation and evidence of improvements in diagnostic pathways, healthcare efficiency, and patient-centred outcomes remain limited. Further implementation research is therefore necessary before broader routine adoption can be recommended [3,6,10,17,18].

### 4.3. Orthopaedic-Specific Risks and Limitations of Artificial Intelligence

Despite the potential of artificial intelligence (AI) to improve diagnostic consistency and support clinical decision-making, its use in orthopaedics introduces risks that extend beyond conventional concerns regarding model accuracy. In musculoskeletal imaging, false-negative results may delay the diagnosis of fractures, implant-related abnormalities, infection, or other clinically important findings, whereas false-positive results may lead to unnecessary additional imaging, consultations, procedures, or changes in treatment. These risks may be amplified when AI systems are applied to uncommon presentations, technically inadequate images, postoperative anatomy, or patient populations that are insufficiently represented in the development datasets. Although several studies have reported high diagnostic performance, particularly for fracture detection and musculoskeletal image analysis, the generalizability of these findings remains limited by heterogeneous datasets, retrospective designs, and insufficient external or multicentre validation [7,8,9,10,11,14,24].

A further concern is automation bias, whereby clinicians may give excessive weight to an algorithmic recommendation or may fail to independently reconsider an AI output. Although the available orthopaedic literature does not always measure automation bias directly, this risk is particularly relevant when AI results are presented with high confidence, when the system is integrated into a time-pressured clinical workflow, or when users have limited information about the model’s training population and known failure modes. In orthopaedics, inappropriate reliance on an AI-supported diagnosis or risk estimate could influence fracture management, surgical indication, implant selection, perioperative planning, or postoperative surveillance. Evidence from AI-assisted hip fracture detection supports the use of these systems as triage or decision-support tools followed by clinician review, rather than as autonomous diagnostic solutions [24]. Accordingly, AI should remain a clinician-supervised decision-support tool, with clearly defined responsibilities for reviewing, accepting, overriding, and documenting algorithmic outputs [4,5,6,18,19].

Dataset-related and device-related biases represent additional important limitations. Orthopaedic AI models may be affected by imbalances in age, sex, ethnicity, body habitus, disease severity, implant type, imaging equipment, acquisition protocols, and healthcare setting. Moreover, differences between hospitals, manufacturers, radiographic systems, scanners, and software environments may alter model performance when an algorithm is transferred from its development setting to routine clinical practice. These factors can reduce generalizability and result in unequal diagnostic performance across patient groups or institutions. The importance of representative datasets and subgroup-specific validation is further underscored by the recognized influence of sex- and gender-related differences on the presentation, pathology, and treatment response of orthopaedic conditions [2,4,5,6,14,19].

The clinical consequences of inappropriate AI-supported decisions may be substantial. A missed fracture or incorrectly classified postoperative image may delay treatment, whereas an erroneous prediction of complication risk may influence admission, surveillance, or operative decision-making. In the context of hip fracture detection, the potential consequences of missed diagnoses reinforce the need for careful clinical review and prospective evaluation before routine deployment [24]. Similarly, inaccurate patient-selection or implant-planning tools may contribute to inappropriate indications, suboptimal implant choice, or avoidable complications [12,17,18]. These risks demonstrate that technical performance alone cannot establish clinical readiness. Within the ORION framework, robust external validation, prospective evaluation, workflow safeguards, clinician oversight, monitoring of subgroup performance, and evidence of patient benefit should therefore be considered important prerequisites for progression toward routine clinical adoption [4,5,6,12,14,17,18,24].

### 4.4. Beyond Diagnostic Accuracy: The Need for Clinical Validation

Historically, research on artificial intelligence in orthopaedics has emphasized algorithmic performance, with most studies reporting diagnostic accuracy, sensitivity, specificity, or the area under the receiver operating characteristic curve as their primary outcomes [3,4]. Although these metrics remain indispensable during algorithm development, they provide only limited evidence of clinical utility.

Successful implementation also requires evidence of reproducibility across institutions, prospective validation, adequate calibration, integration into clinical workflows, clinician acceptance, patient safety, and improvements in clinically meaningful outcomes. Importantly, these implementation domains were consistently identified across virtually all contemporary reviews included in the present synthesis [6,17,19]. Therefore, future research on artificial intelligence in orthopaedics should progressively shift from optimizing computational performance toward generating implementation evidence capable of supporting safe and widespread clinical adoption. Such evidence should include external and prospective validation, assessment of workflow integration, monitoring of subgroup performance, clinician oversight, and evaluation of patient-centred outcomes.

### 4.5. Translating Artificial Intelligence into Routine Orthopaedic Practice

The principal conceptual contribution of the present review is the development of the ORION Clinical Readiness Framework.

Unlike previous reviews that primarily summarize isolated AI applications, ORION proposes an evidence-informed model describing the sequential stages required for successful translation of AI into routine orthopaedic practice [17,18].

**Figure 4:**
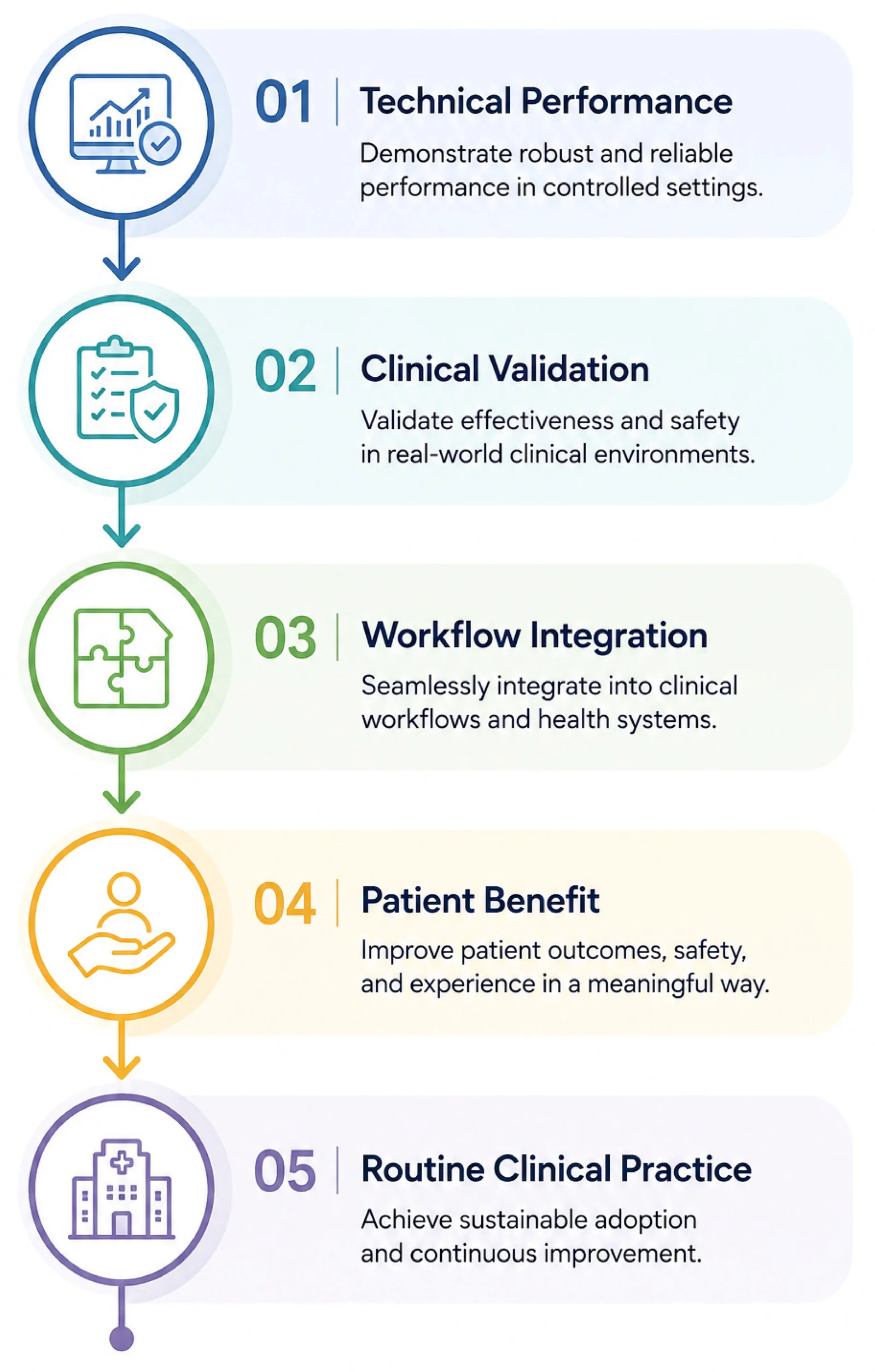
The ORION Clinical Readiness Framework.

Figure 4 presents an evidence-informed conceptual framework describing the sequential transition of artificial intelligence from technological innovation to routine clinical adoption in orthopaedics. The ORION framework comprises five sequential domains of clinical readiness: Technical Performance, Clinical Validation, Workflow Integration, Patient Benefit, and Routine Clinical Adoption. The broader translational pathway illustrated in Figure 1 includes an antecedent innovation and algorithm-development phase, which precedes the five ORION domains but is not itself an ORION domain. These domains are also intended to capture safety-related requirements, including the identification of failure modes, assessment of subgroup performance, clinician oversight, and monitoring of unintended consequences during clinical deployment.

As illustrated in Figure 4, the successful implementation of artificial intelligence extends far beyond algorithm development. Technical performance represents the initial ORION domain after the antecedent innovation and algorithm-development phase. Sustainable clinical adoption requires progressive evidence of external validation, integration into routine clinical workflows, demonstrable patient benefit, and continuous quality improvement. Across the included literature, artificial intelligence technologies were therefore evaluated according to their progression through these interconnected domains. Importantly, these domains should not be interpreted as isolated milestones but rather as components of a continuous translational pathway from technological development to routine clinical adoption.

Excellent diagnostic performance alone is insufficient to justify clinical implementation [4,6,19]. Successful adoption also requires evidence that artificial intelligence systems maintain their performance across diverse patient populations, integrate efficiently into existing clinical workflows, improve clinical decision-making, generate measurable patient benefit, and satisfy applicable ethical and regulatory requirements.

The ORION framework therefore provides a practical, conceptual roadmap to support clinicians, researchers, healthcare organizations, regulatory agencies, and industry partners in evaluating implementation maturity and prioritizing future research. Rather than functioning as a quantitative scoring system, ORION should be understood as an evidence-informed translational framework that describes how artificial intelligence in orthopaedics progresses from innovation toward routine clinical adoption.

### 4.6. Toward Precision Orthopaedics

One of the most important observations emerging from this review is the progressive evolution of orthopaedic AI from isolated diagnostic algorithms toward integrated precision musculoskeletal care [4,6].

Early AI applications were primarily designed to address single clinical problems, such as fracture detection, osteoarthritis grading, implant recognition, or image segmentation. More recent approaches have begun to integrate multiple complementary sources of patient information, including radiographs, CT and MRI examinations, electronic health records, laboratory findings, biomechanical parameters, wearable-sensor data, and patient-reported outcomes. This development represents a gradual shift from single-task and single-modality systems toward multimodal models that may support more comprehensive diagnostic, prognostic, and treatment-planning decisions across the orthopaedic care pathway [4,6,10,17,18].

However, this progression remains largely conceptual or at an early stage of clinical translation. Multimodal systems continue to face important challenges related to data harmonization, interoperability, missing data, external validation, interpretability, and integration into clinical workflows [4,6,17,18].

This evolution reflects a broader paradigm shift within orthopaedics. Rather than supporting isolated diagnostic decisions, AI is progressively becoming part of a comprehensive digital ecosystem capable of assisting clinicians throughout the patient journey [17,18].

In this context, the future of orthopaedic AI should not be viewed simply as the development of “smarter algorithms,” but rather as the development of intelligent clinical ecosystems capable of integrating multiple sources of patient-specific information into individualized diagnostic, prognostic, and therapeutic strategies [4,6,19].

Emerging technologies—including digital twins, federated learning, explainable AI (XAI), large multimodal foundation models, smart implants, wearable monitoring, and continuous-learning systems—may further support this transition, although their clinical utility and implementation remain to be established [4,6,17,18].

If successfully validated and responsibly implemented, these technologies may contribute to a transition toward precision orthopaedics, in which diagnostic, prognostic, and therapeutic decisions are increasingly informed by integrated, patient-specific data.

### 4.7. Clinical Implications

The findings of the present review have several important implications for clinical practice [3,4,17].

First, AI should currently be regarded as a clinical augmentation technology rather than an autonomous decision-maker [7,10].

Across virtually every orthopaedic subspecialty, the literature consistently supports the role of AI in improving diagnostic consistency, reducing variability, increasing workflow efficiency, and assisting clinicians in complex decision-making. Nevertheless, none of the contemporary evidence supports replacing orthopaedic surgeons or musculoskeletal radiologists with autonomous AI systems [6,19].

Second, the maturity of AI applications differs substantially across orthopaedic domains. Fracture detection and selected musculoskeletal imaging applications have accumulated comparatively stronger evidence for carefully supervised implementation, particularly when AI is used in combination with clinician assessment [7,8,10,21]. In contrast, multimodal decision-support systems, rehabilitation technologies, digital twins, predictive analytics, and autonomous surgical guidance remain supported mainly by developing, retrospective, proof-of-concept, or early implementation evidence [4,5,6,12,17,18].

Third, implementation strategies should emphasize clinician supervision, external validation, interoperability, explainability, appropriate governance, and prospective evaluation of patient-centred outcomes before routine adoption [4,5,6,12,14,17,18,19]. These priorities were recurrently identified across the included evidence syntheses and are summarized in Table 5.

Finally, healthcare institutions considering the implementation of AI should recognize that successful adoption depends not only on algorithmic performance but also on organizational readiness, digital infrastructure, multidisciplinary collaboration, governance frameworks, and continuous monitoring of model performance [4,5,6,17,18].

Regulatory, medico-legal, and economic considerations are also relevant to the responsible implementation of orthopaedic AI. Depending on its intended use and risk profile, an AI-enabled medical device may require regulatory assessment or authorization under applicable national or regional frameworks. However, regulatory authorization alone does not establish clinical effectiveness, long-term safety, cost-effectiveness, or suitability for every healthcare environment. Clear allocation of professional and medico-legal responsibility is also important when clinicians rely on, override, or disagree with AI-generated recommendations. Economic evaluations should additionally consider acquisition and maintenance costs, infrastructure requirements, staff training, workflow effects, downstream testing, healthcare utilization, and patient-centred value. These aspects should be incorporated into prospective implementation studies rather than evaluated only after deployment [4,5,17,18].

### 4.8. Strengths

The present review possesses several important strengths. Unlike many previous reviews that have focused on isolated artificial intelligence applications or individual orthopaedic subspecialties, the present study adopts a translational perspective by evaluating artificial intelligence across the orthopaedic patient care pathway [3,4,5,6,7,8,9,10,11,12,13,14,15,16,17,18,19].

This review synthesizes evidence from 21 contemporary evidence syntheses and organizes the available literature according to clinical pathway, implementation maturity, barriers to translation, and patient-centred outcomes. The evidence was structured into complementary analytical domains, including the characteristics of the evidence base, the orthopaedic patient pathway, the overall evidence landscape, clinical readiness, barriers to implementation, and recommendations for clinical implementation. This structure facilitated comparison among heterogeneous artificial intelligence applications while maintaining a focus on implementation rather than isolated algorithmic performance.

The principal contribution of this review is the development of the ORION Clinical Readiness Framework, an original, evidence-informed conceptual model that integrates recurring implementation themes identified across the contemporary literature [4,5,6,17,18,19].

### 4.9. Limitations

Several limitations should be acknowledged. First, the present study represents a structured qualitative evidence synthesis rather than a quantitative meta-analysis. Consequently, pooled estimates of diagnostic accuracy were intentionally not calculated because of marked heterogeneity in AI methodologies, orthopaedic applications, validation strategies, and reported outcome measures.

Second, the available literature remains dominated by retrospective investigations and internally validated AI models, whereas prospective, multicentre implementation studies remain relatively uncommon. Considerable variability also exists in reporting standards, external validation methodologies, AI architectures, and clinical outcome measures, limiting direct comparisons between studies.

Third, the ORION Clinical Readiness Framework represents an evidence-informed conceptual framework derived from recurring implementation patterns rather than a prospectively validated scoring instrument. Future studies should evaluate its reproducibility, interobserver reliability, and applicability across different healthcare systems and orthopaedic subspecialties.

The evidence base may also be affected by publication bias, as studies reporting favourable technical performance or successful implementation may be more likely to be published than negative, inconclusive, or unsuccessful studies. In addition, the rapid evolution of artificial intelligence technologies limits the durability of the available evidence, as models, platforms, regulatory conditions, and implementation practices may change substantially over relatively short periods. Randomized implementation studies evaluating patient-centred outcomes, such as functional recovery, quality of life, safety, healthcare utilization, and cost-effectiveness, also remain scarce. These limitations require cautious interpretation of reported clinical readiness and reinforce the need for longitudinal evaluation of AI technologies in real-world healthcare settings.

Although regulatory-cleared or certified AI software and evidence regarding clinical implementation are highly relevant to the translation of AI frameworks into practice, their systematic assessment was beyond the scope of the present work. Such an assessment would require a dedicated analysis of regulatory databases, product-specific indications, certification pathways, software versions, and real-world implementation evidence. Moreover, including a comprehensive overview of currently available AI products would shift the primary focus of this manuscript from the methodological development of ORION toward a broader review of the regulatory and product landscape. Therefore, these aspects were not systematically evaluated in the present study and should be addressed in dedicated future investigations.

### 4.10. Future Directions

The evidence synthesized in the present review consistently suggests that future orthopaedic AI research should progressively shift from improving algorithmic performance toward improving clinical implementation [4,6].

Several priorities emerged repeatedly across the included literature [19]. First, prospective multicentre validation should become the minimum standard before widespread clinical adoption [17,18]. Second, future AI systems should incorporate explainable AI methodologies capable of improving clinician confidence, facilitating regulatory approval, and enhancing transparency [4,6]. Third, interoperability between AI platforms and electronic health records, Picture Archiving and Communication Systems (PACS), robotic platforms, and wearable technologies should become a major research priority. Fourth, future investigations should increasingly evaluate patient-centred outcomes, including functional recovery, quality of life, complication rates, healthcare utilization, patient satisfaction, and cost-effectiveness. Finally, international collaboration will be essential for developing globally representative datasets capable of reducing algorithmic bias, improving external validity, and facilitating equitable implementation across diverse healthcare systems [19].

Future studies should also apply the ORION framework to systematically evaluate regulatory-cleared or certified AI software in orthopaedic and musculoskeletal care, integrating regulatory status, intended clinical use, external validation, interoperability, and real-world implementation evidence.

## 5. Conclusions

Artificial intelligence has rapidly evolved from an experimental computational technology into a clinically relevant component of contemporary orthopaedic research, with applications extending across the patient care pathway, including diagnosis, surgical planning, intraoperative guidance, postoperative monitoring, rehabilitation, and long-term outcome prediction.

Among the applications currently reported, musculoskeletal imaging appears to represent the most clinically mature domain. In contrast, predictive analytics, multimodal decision-support systems, robotic platforms, rehabilitation technologies, and foundation models remain at earlier stages of clinical translation despite encouraging technical performance.

The present review indicates that the principal challenge facing orthopaedic artificial intelligence is no longer algorithm development alone, but its rigorous translation into clinical practice. Limited external validation, heterogeneous datasets, workflow-integration difficulties, limited interpretability, regulatory uncertainty, ethical and governance challenges, and insufficient prospective evidence of meaningful patient benefit continue to constrain widespread implementation.

To help conceptualize this translational gap, we propose the ORION Clinical Readiness Framework as an evidence-informed five-domain conceptual model describing a potential progression from Technical Performance through Clinical Validation, Workflow Integration, and Patient Benefit to Routine Clinical Adoption. The innovation and algorithm-development phase precedes these five domains and is illustrated separately in Figure 1. ORION is not intended to function as a validated clinical, regulatory, or quantitative implementation tool. Rather, it is proposed as a preliminary framework that may help organize discussions of implementation maturity, identify potential translational barriers, and support the formulation of future research questions. Its structure, applicability, and validity require prospective evaluation and refinement across different orthopaedic settings before it can be considered an established implementation framework.

Future advances in orthopaedic artificial intelligence will depend not only on increasingly sophisticated algorithms, but also on robust multicentre validation, standardized implementation strategies, explainable and trustworthy AI, responsible governance, interoperability with healthcare systems, and demonstration of measurable improvements in patient-centred outcomes.

Ultimately, the future of orthopaedic artificial intelligence will be determined not only by how accurately algorithms perform in experimental environments, but by whether they can reliably improve clinical decision-making, patient outcomes, and healthcare delivery in everyday practice.

## Figures and Tables

**Table 1:** Evidence Synthesis of Artificial Intelligence Applications in Orthopaedics.

Study	Evidence Type	Clinical Domain	Primary AI Application	Patient Pathway	Key Findings	Main Barrier	ORION Clinical Readiness Stage
Kuo et al., 2022 [21]	Systematic Review + Meta-analysis	Trauma	Fracture detection	Diagnosis	Pooled accuracy did not differ significantly from that of clinicians reading the same images; the clearest gain came from pairing algorithm and reader rather than substituting one for the other.	Few prospective or externally validated studies.	Early Clinical Adoption
Gupta et al., 2023 [16]	Systematic Review	Shoulder Surgery	Outcome prediction	Planning/Surgery	Applications covered implant identification, rotator cuff pathology and complication risk, but the authors judged the field to fall well short of clinical usability.	External testing almost entirely absent.	Developing
okHusarek et al., 2024 [7]	Systematic Review + Meta-analysis	Trauma	Commercial fracture detection	Diagnosis	Across marketed products accuracy was high and rose further when algorithm and reporting clinician were combined; funding source was tabulated for every primary study.	Independent replication; heterogeneous datasets.	Early Clinical Adoption
Hansen et al., 2024 [8]	Systematic Review + Meta-analysis	Wrist fractures	Automated fracture detection	Diagnosis	On wrist radiographs, deep-learning models matched the reading performance of experts.	Few multicentre datasets.	Early Clinical Adoption
Gitto et al., 2024 [9]	Narrative Review	Musculoskeletal Imaging	Image interpretation	Diagnosis	Gains were reported for fracture pick-up, implant assessment, bone-age estimation and osteoarthritis grading, alongside shorter radiology turnaround.	External and prospective evidence still sparse.	Developing
Geda et al., 2024 [3]	Systematic Review + Meta-analysis	General Orthopaedics	Surgical AI	Entire pathway	Applicability was broad across surgical orthopaedics, although outcome evidence remained thin.	Patient-outcome evidence not established.	Developing
Georgiakakis et al., 2024 [18]	Narrative Review	Planned Orthopaedic Care	Elective care pathway	Entire pathway	Elective pathways showed potential at every step—planning, robotics and remote follow-up—conditional on infrastructure being in place.	Implementation infrastructure; external testing.	Developing
Mohammed et al., 2024 [19]	Systematic Review	Disease Detection	Trustworthy AI	Diagnosis	Detection performance was strong, but the taxonomy foregrounded explainability, fairness and trustworthiness as conditions of deployment.	Transparency, governance, ethical assurance.	Emerging
Dijkstra et al., 2024 [14]	Systematic Review	Orthopaedic Trauma	Machine-learning prediction models	Patient Selection/Prognosis	Discrimination in trauma prediction models was moderate to good; reporting quality and external testing were frequently deficient.	Deficient reporting; no external testing.	Emerging
Tafat et al., 2024 [5]	Comprehensive Review	General Orthopaedics	Innovation across the surgical pathway	Entire pathway	Coverage extended from imaging to intraoperative guidance and prognosis, with interpretability and data stewardship treated as the decisive issues.	Interpretability; data quality; stewardship.	Emerging
Longo et al., 2025 [10]	Systematic Review	Orthopaedic Imaging	Multimodal imaging	Diagnosis/Planning	Performance held across MRI, CT, radiography and ultrasound, most convincingly for segmentation and automated measurement.	Heterogeneous methods; no randomised evidence.	Developing
Oettl et al., 2025 [11]	Narrative Review	Musculoskeletal Imaging	AI-assisted imaging	Diagnosis	Reported benefits were operational as much as diagnostic: faster throughput and more reproducible reads.	Validation; interpretability; workflow fit.	Developing
Tian et al., 2025 [15]	Systematic Review + Meta-analysis	Arthroplasty	Surgical indication	Treatment Planning	Candidates for total knee arthroplasty were identified accurately by machine-learning models.	Database quality; no prospective testing.	Developing
Schneller et al., 2025 [13]	Systematic Review	Shoulder Arthroplasty	Predictive analytics	Planning/Surgery	Predictive performance for shoulder arthroplasty outcomes was moderate to good; reporting was too sparse to permit replication.	Reporting insufficient for reproduction.	Developing
Han et al., 2025 [4]	Comprehensive Review	General Orthopaedics	Precision Orthopaedics	Entire pathway	Coverage spanned diagnosis, robotics, rehabilitation and multimodal decision-making within a precision-orthopaedics framing.	Fragmented data; interoperability; oversight.	Emerging
Sharma et al., 2025 [12]	Structured Narrative Review	Orthopaedic Surgery	Postoperative prediction	Surgery/Follow-up	For postoperative complications, machine-learning models exceeded conventional regression-based risk tools.	Validation; calibration; interpretability.	Developing
Banskota et al., 2025 [23]	Systematic Review	Education	AI-assisted training	Education	Training benefits included better simulation, objective assessment of technical skill and individualised curricula.	No longitudinal data on transfer to practice.	Emerging
Zhang et al., 2025 [20]	Systematic Review	Knee Arthroplasty	X-ray analysis	Postoperative Imaging	On postoperative knee radiographs, models identified implant type, alignment, loosening and periprosthetic joint infection.	Larger multicentre datasets required.	Developing
Luo et al., 2026 [6]	Narrative Review	Multimodal Imaging	Multimodal AI	Entire pathway	Combining imaging with clinical and biochemical data was positioned as the next phase of precision musculoskeletal care.	Generalisability; fit with existing workflows.	Emerging
Dwajan et al., 2026 [17]	Structured Narrative Review	General Orthopaedics	Clinical decision support	Entire pathway	Reach extended across the whole pathway, yet deployment lagged well behind reported capability.	Validation gap; regulation; clinician trust.	Emerging
Young, 2026 [22]	Narrative Review	Orthopaedic Trauma	Trauma ecosystem	Entire pathway	Scope had widened beyond fracture pick-up into training, administrative workload and postoperative surveillance.	Validation; oversight; workflow fit.	Emerging

Abbreviation: AI, artificial intelligence.

**Table 2:** Artificial Intelligence Across the Orthopaedic Patient Care Pathway.

Patient Pathway Stage	Representative Studies	Main AI Applications	Principal Clinical Contribution	Current Implementation Status *
Diagnosis	Kuo 2022 [21]; Husarek 2024 [7]; Hansen 2024 [8]; Gitto 2024 [9]; Longo 2025 [10]; Oettl 2025 [11]	Fracture detection, image segmentation, osteoarthritis grading, implant recognition, image enhancement.	High diagnostic accuracy, reduced missed fractures, improved workflow efficiency and diagnostic consistency.	Early Implementation in Selected Settings
Patient Selection & Risk Stratification	Tian 2025 [15]; Gupta 2023 [16]; Dijkstra 2024 [14]; Sharma 2025 [12]	Surgical indication, complication prediction, outcome prediction, mortality prediction.	Improved patient selection and perioperative risk assessment.	Developing
Preoperative Planning	Tafat 2024 [5]; Han 2025 [4]; Geda 2024 [3]; Georgiakakis 2024 [18]	3D reconstruction, implant planning, surgical simulation, robotics planning.	More personalized surgical planning and improved operative precision.	Developing
Intraoperative Support	Han 2025 [4]; Geda 2024 [3]; Georgiakakis 2024 [18]; Dwajan 2026 [17]	Robotic surgery, computer vision, navigation systems, augmented reality.	Improved implant positioning, navigation and operative assistance.	Developing
Postoperative Monitoring	Zhang 2025 [20]; Sharma 2025 [12]; Young 2026 [22]; Georgiakakis 2024 [18]	Implant surveillance, complication prediction, remote monitoring.	Earlier complication detection and optimized postoperative surveillance.	Developing
Rehabilitation & Long-term Follow-up	Luo 2026 [6]; Young 2026 [22]; Dwajan 2026 [17]	Wearable monitoring, multimodal AI, rehabilitation platforms, smart implants.	Personalized rehabilitation and longitudinal outcome monitoring.	Emerging
Education & Research ^†^	Banskota 2025 [23]	Virtual reality, adaptive learning, AI-assisted simulation.	Improved surgical training, objective skills assessment and personalized education.	Emerging

Abbreviation: AI, artificial intelligence. * Implementation status classified according to the ORION Clinical Readiness Framework. ^†^ Education and research were included as cross-cutting applications rather than as sequential stages of the patient care pathway.

**Table 3:** ORION Clinical Readiness Framework for Artificial Intelligence Applications in Orthopaedics.

Clinical Domain	Technical Performance	External Validation	Workflow Integration	Patient Outcome Evidence	Overall Clinical Readiness
Fracture Detection	Excellent	High	High	Moderate	Potential Early Adoption in Selected Settings
Musculoskeletal Imaging	Excellent	Moderate	High	Limited	Developing
Knee Arthroplasty Imaging	Excellent	Moderate	High	Limited	Potential Early Adoption in Selected Settings
Shoulder Arthroplasty	Good	Limited	Moderate	Moderate	Developing
Orthopaedic Trauma Prediction Models	Good	Limited	Limited	Limited	Emerging
Postoperative Complication Prediction	Excellent	Limited	Moderate	Moderate	Developing
General Orthopaedic Surgery	Excellent	Moderate	High	Moderate	Developing
Elective Orthopaedic Care	Good–Excellent	Limited	High	Moderate	Developing
Multimodal Imaging	Excellent	Limited	High	Moderate	Developing
Education & Surgical Training	Good–Excellent	Limited	Moderate	Indirect	Developing

Operational criteria for Table 3. Each domain was rated across four dimensions by two assessors (R.D.N.G. and P.L.M.), with disagreements resolved by consensus. The rating anchors were defined a priori as follows. Technical Performance (reported diagnostic or predictive accuracy of the AI models): *Excellent* = pooled or predominant accuracy/AUC ≥ 0.90 or sensitivity and specificity ≥ 90%; Good = 0.80–0.89 or 80–89%; Moderate = 0.70–0.79; Limited ≤ 0.70 or inconsistent. External Validation: High = multiple independent multicentre external validations reported; Moderate = at least one external validation; Limited = predominantly internal validation only. Workflow Integration: *High* = evidence of integration into routine clinical systems (e.g., PACS, EHR) or commercial deployment; Moderate = pilot or single-centre integration; Limited = research/experimental settings only. Patient Outcome Evidence: Moderate = comparative or prospective evidence of clinical benefit; Limited = surrogate or accuracy endpoints only; Indirect = benefit inferred rather than measured. Potential Early Adoption in Selected Settings = excellent technical performance with high external validation and evidence of workflow integration in selected institutions or commercial environments; this designation does not imply widespread or homogeneous routine adoption.

**Table 4:** Major Barriers to Clinical Translation of Artificial Intelligence in Orthopaedics.

Barrier	Evidence from the Literature	Representative Studies	Impact on Clinical Implementation
Limited external validation	Most AI models remain internally validated, with few multicentre prospective studies.	Kuo [21], Husarek [7], Dijkstra [14], Gupta [16], Schneller [13], Sharma [12], Luo [6], Young [20	Reduces confidence, generalizability and routine clinical adoption.
Methodological heterogeneity	Variation in datasets, AI architectures, outcome definitions and reporting standards limits comparison across studies.	Longo [10], Hansen [8], Zhang [20], Tian [15], Geda [3]	Prevents reproducibility and robust evidence synthesis.
Workflow integration	Limited interoperability with PACS, electronic health records and hospital information systems.	Georgiakakis [18], Tafat [5], Sharma [12], Han [4], Luo [6]	Restricts implementation in routine clinical workflows.
Limited patient-centred evidence	Most studies report diagnostic performance rather than functional outcomes, quality of life or cost-effectiveness.	Geda [3], Sharma [12], Longo [10], Schneller [13]	Uncertainty regarding real clinical benefit.
Algorithm explainability (Black-box AI)	Poor transparency limits clinician confidence and regulatory acceptance.	Mohammed [19], Han [4], Tafat [5], Oettl [11]	Delays clinician acceptance and regulatory approval.
Ethical and regulatory challenges	Privacy, data governance, legal responsibility and algorithmic bias remain unresolved.	Mohammed [19], Han [4], Young [22], Tafat [5]	Limits widespread implementation across healthcare systems.
Data quality and interoperability	Fragmented databases, inconsistent annotations and poor dataset diversity impair model robustness.	Luo [6], Han [4], Tian [15], Tafat [5]	Restricts external validation and multimodal AI development.
Implementation gap	Excellent technical performance has not translated into routine clinical practice.	Dwajan [17], Luo [6], Georgiakakis [18], Han [4]	Represents the principal challenge identified across the contemporary literature.

**Table 5:** Evidence-based recommendations for clinical implementation of artificial intelligence in orthopaedics.

Recommendation	Supporting Evidence	Expected Clinical Impact
Implement AI as a clinical decision-support tool rather than an autonomous system.	Husarek [7]; Kuo [21]; Han [4]; Oettl [11]; Sharma [12]	Improves diagnostic accuracy while preserving clinician oversight and patient safety.
Require robust multicentre external validation before routine implementation.	Husarek [7]; Dijkstra [14]; Sharma [12]; Young [22]; Gupta [16]; Tian [15]	Increases model generalizability and confidence across different healthcare settings.
Integrate AI into existing clinical workflows and digital infrastructure (e.g., PACS and electronic health records).	Geda [3]; Han [4]; Luo [6]; Dwajan [17]	Reduces disruption to clinical routine and supports sustainable, scalable adoption.
Establish multidisciplinary governance, regulatory compliance, and continuous performance monitoring.	Han [4]; Georgiakakis [18]; Mohammed [19]; Sharma [12]	Ensures patient safety, accountability, and maintenance of model performance over time.

## Data Availability

No new data were created or analyzed in this study. Data sharing is not applicable to this article.
